# More Than Just a Plaque: A Diagnostic Challenge in a Case of Suspected Cutaneous Localized Nodular Amyloidosis

**DOI:** 10.1002/ccr3.71514

**Published:** 2025-11-20

**Authors:** Sandesh Shah, Joshana Shrestha

**Affiliations:** ^1^ Department of Dermatology Nepal Medical College and Teaching Hospital Kathmandu Nepal

**Keywords:** amyloid, localized amyloidosis, PCLNA, primary cutaneous localized nodular amyloidosis

## Abstract

Primary Localized Cutaneous Nodular Amyloidosis (PLCNA) can rarely present on the face and may mimic more common dermatoses. In such atypical cases, especially in resource‐limited settings where confirmatory tests like Congo Red staining are unavailable, histopathological examination is crucial for diagnosis. Early recognition is vital for proper management and monitoring for potential systemic involvement.

## Graphical abstract

A solitary facial nodular plaque clinically mimicking common dermatoses, diagnosed as Primary Localized Cutaneous Nodular Amyloidosis (PLCNA) through histopathology in a resource‐limited setting.

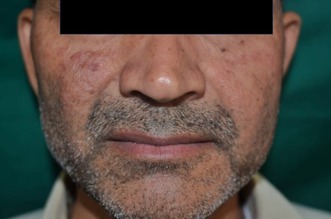

## 1 Introduction

Amyloidosis encompasses a diverse range of disorders defined by the extracellular accumulation of insoluble fibrillar proteins known as amyloid in various tissues and organs. These deposits can impair the function of the affected structures [1]. Amyloidosis is generally categorized into systemic and localized forms, with the localized type confined to specific organs or tissues without systemic impact. Primary localized cutaneous amyloidosis (PLCA) represents a form of localized amyloidosis where amyloid accumulation is restricted to the skin. There are three clinical variants of PLCA: macular, lichen, and nodular amyloidosis, with nodular being the least common subtype [2, 3].

PLCNA typically manifests as single or multiple firm, waxy nodules or plaques of yellow or reddish‐brown. These lesions usually occur on the extremities, face, or trunk, but can arise anywhere on the skin [4, 5]. The nodular form shows distinct histopathological features compared to macular and lichen variants, as the amyloid deposits primarily consist of immunoglobulin light chains (AL type) produced by a localized monoclonal plasma cell population [2, 6].

Histological confirmation is critical for diagnosis, with Congo red staining revealing the characteristic apple‐green birefringence under polarized light. Immunohistochemistry commonly shows kappa or lambda light chain restriction, indicating localized plasma cell dyscrasia [7, 8].

While PLCNA typically has an indolent course, there are rare cases of progression to systemic amyloidosis. Therefore, a comprehensive evaluation to exclude systemic involvement and regular follow‐ups are recommended [9, 10]. We present a case of PLCNA that highlights its clinical features, diagnostic processes, and monitoring strategies.

## 2 Case History/Examination

A 42‐year‐old male patient visited the dermatology outpatient department with a solitary, non‐itchy, painless, reddish‐brown elevated lesion on the right cheek that had been gradually increasing since the last year. There was no previous history of trauma, discharge, ulceration, photosensitivity, or systemic symptoms like fever, shortness of breath, weight loss, fatigue, or joint pain.

**FIGURE 1:**
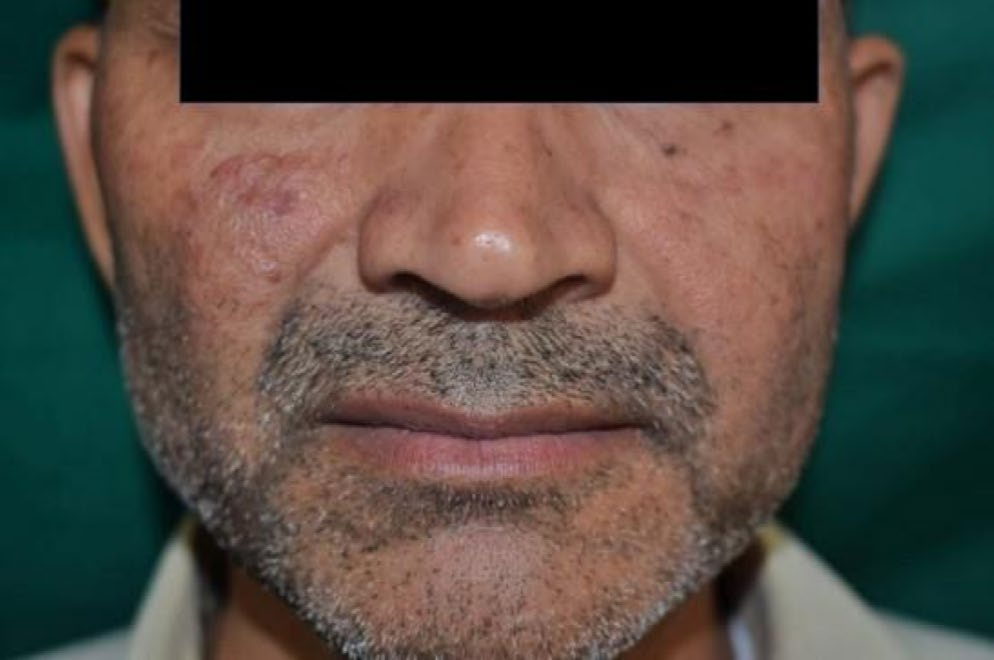
Well‐defined to ill‐defined, slightly elevated, shiny erythematous nodular plaque in the right malar region, approximately 2.5 × 1.5 cm in diameter, with a smooth, mildly indurated surface.

**FIGURE 2:**
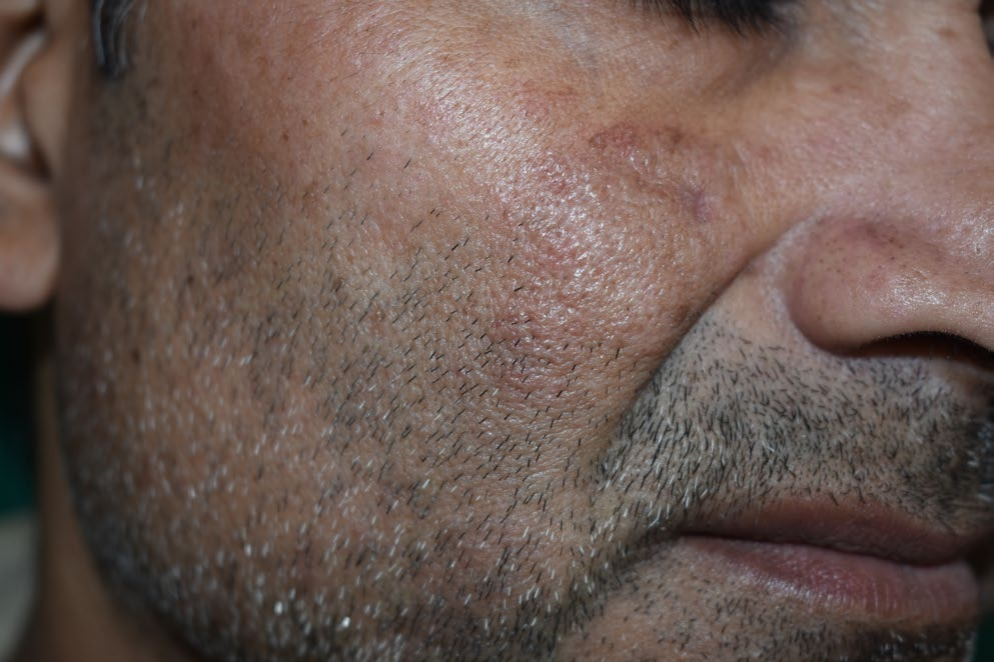
Well‐defined to ill‐defined, slightly elevated, shiny erythematous plaque in the right malar region, approximately 2.5 × 1.5 cm in diameter, with a smooth, mildly indurated surface.

Cutaneous examination revealed a well‐defined to ill‐defined, slightly elevated, shiny erythematous nodular plaque in the right malar region, approximately 2.5 × 1.5 cm in diameter, with a smooth, mildly indurated surface and without scales, ulceration, or crusting (Figures 1 and 2). No history of similar lesions in other areas. No regional lymphadenopathy was noted. The remaining mucocutaneous and systemic examination was normal.

## 3 Differential Diagnosis, Investigations, and Treatment

**FIGURE 3:**
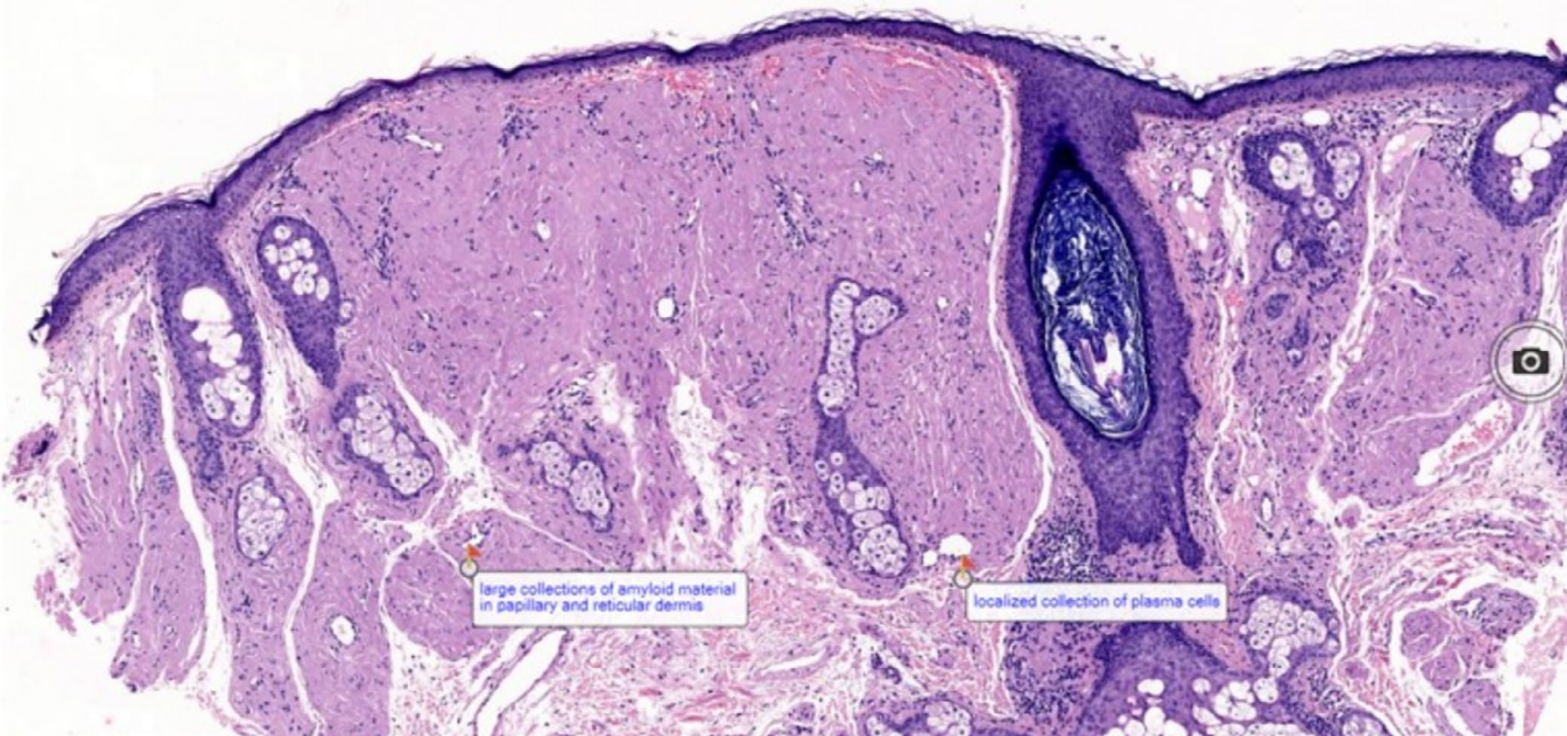
Showing large collections of amyloid material in papillary and reticular dermis and a localized collection of plasma cells.

Routine laboratory investigations, including complete blood count, liver and renal function tests, urinalysis, chest x‐ray, and abdominal ultrasound, were within normal limits. ANA (antinuclear antibody) and serum protein electrophoresis were negative. Punch biopsy was done, and histopathological examination revealed thinned epithelium with no evidence of ulceration or epidermotropism. Homogenous eosinophilic amorphous deposits were present extensively in the papillary and reticular dermis, replacing the normal dermal collagen. Focal infiltrates of plasma cells were observed in the dermis, predominantly at the periphery of amyloid deposits. Skin appendages such as hair follicles and sebaceous glands appear preserved in areas away from dense amyloid deposition, with mild perivascular lymphoplasmacytic infiltrates in parts (Figure 3). Congo Red Stain and Immunohistochemistry tests were unavailable, so they could not be done. In the absence of these special stains, differential diagnoses such as colloid milium should also be considered. Simple stains like PAS and PAS‐D can help in distinguishing between amyloid and colloid deposits; however, these were not available in our setting. Dermoscopy, which could have provided additional diagnostic support, was not performed in this case due to unavailability. The patient was counseled regarding the disease and its nature, and supportive management was advised. However, he has not been on follow‐up, and therefore the long‐term outcome and systemic evaluation could not be assessed.

## 4 Conclusion

The rarity of PLCNA on the face, combined with its clinical resemblance to more common dermatoses, underscores the importance of considering a broad differential diagnosis. In resource‐limited settings, where confirmatory tools like Congo Red staining may be unavailable, histopathological evaluation becomes essential for accurate diagnosis. Maintaining a high index of suspicion and recognizing key histological features are critical. Early diagnosis not only facilitates appropriate local management but also enables long‐term monitoring, given the potential—albeit rare—risk of progression to systemic amyloidosis.

## 5 Discussion

Primary Localized Cutaneous Nodular Amyloidosis (PLCNA) is a rare variant of cutaneous amyloidosis, constituting the least prevalent form, representing approximately 1.5% of all primary cutaneous amyloidosis cases [1]. It is marked by amyloid deposits that arise from immunoglobulin light chains (AL type), produced by localized plasma cell infiltrates [2, 3]. Clinically, it manifests as firm, waxy nodules or plaques that are skin‐colored to reddish‐brown, typically located on the extremities, trunk, or face. Facial involvement, as evidenced in our patient, is exceedingly uncommon and can resemble several dermatological disorders, complicating clinical diagnosis significantly.

Due to the subtle clinical presentation, differential diagnoses included sebaceous hyperplasia, cutaneous sarcoidosis, granuloma faciale, and Jessner's lymphocytic infiltrate (JLI). These conditions exhibit overlapping clinical features. Sebaceous hyperplasia appears as small, soft, yellowish papules on the face, frequently with central umbilication. In contrast to PLCNA, these lesions are not indurated and do not form plaques. Histological examination of sebaceous hyperplasia reveals enlarged sebaceous lobules connected to a central dilated follicular duct, and it lacks both amyloid deposition and plasma cell infiltrates [7]. Cutaneous sarcoidosis may present as indurated plaques or papules on the face, but is usually associated with non‐caseating granulomas accompanied by systemic involvement, which was absent in our patient [4]. Granuloma faciale is another mimic, manifesting as reddish‐ brown plaques primarily on the face. However, histologically, it shows a dense dermal infiltrate featuring a Grenz zone and eosinophils, which were absent in our biopsy [5]. JLI are also present in the facial area, exhibiting interface dermatitis and perivascular lymphocytic infiltrates sans amyloid material, thus aiding in their exclusion from histopathological considerations [6].

The histopathological examination revealed amorphous eosinophilic deposits and plasma cell infiltrates consistent with, but not definitive for, nodular amyloidosis. Given the unavailability of Congo red and immunohistochemistry, a diagnosis of localized cutaneous amyloidosis‐like lesion was favored. We also acknowledge that the histopathology slide provided could have been clearer in demonstrating these deposits. Although Congo red staining and immunohistochemistry for kappa and lambda light chains are essential for confirming amyloid and assessing clonality, we could not conduct these tests due to resource limitations. Typically, Congo red staining displays apple‐green birefringence under polarized light, while immunohistochemistry provides greater specificity, especially in cases of uncertainty [8, 9]. This limitation also means that other diagnoses, including colloid milium, cannot be completely excluded without PAS or PAS‐D staining.

Our findings correlate with earlier reports of facial PLCNA, particularly those by Haran et al., who noted a similar diagnostic challenge where a cheek plaque was initially misdiagnosed as granuloma faciale [10]. Similarly, Woollons and Black highlighted that delayed diagnosis is common in most PLCNA cases because of its similarity to other inflammatory dermatoses [11]. This underscores the diagnostic challenge and reinforces the significance of histopathology in differentiating nodular amyloidosis from its mimics.

## Author Contributions

**Sandesh Shah:** conceptualization, project administration, resources, writing – original draft, writing – review and editing. **Joshana Shrestha:** conceptualization, project administration, resources, writing – original draft, writing – review and editing.

## Funding

The authors have nothing to report.

## Consent

Written informed consent was obtained from the patient for both treatment and publication of clinical information and images. The authors will retain the original signed consent form and provide it to the Publisher if requested.

## Conflicts of Interest

The authors declare no conflicts of interest.

## Data Availability

The authors have nothing to report.
